# Supplementary figures and images for: The use of culturally adapted and translated depression screening questionnaires with South Asian haemodialysis patients in England
Source: PLoS One. 2023 Apr 7;18(4):e0284090. doi: 10.1371/journal.pone.0284090 (PMC10081747; doi:10.1371/journal.pone.0284090)

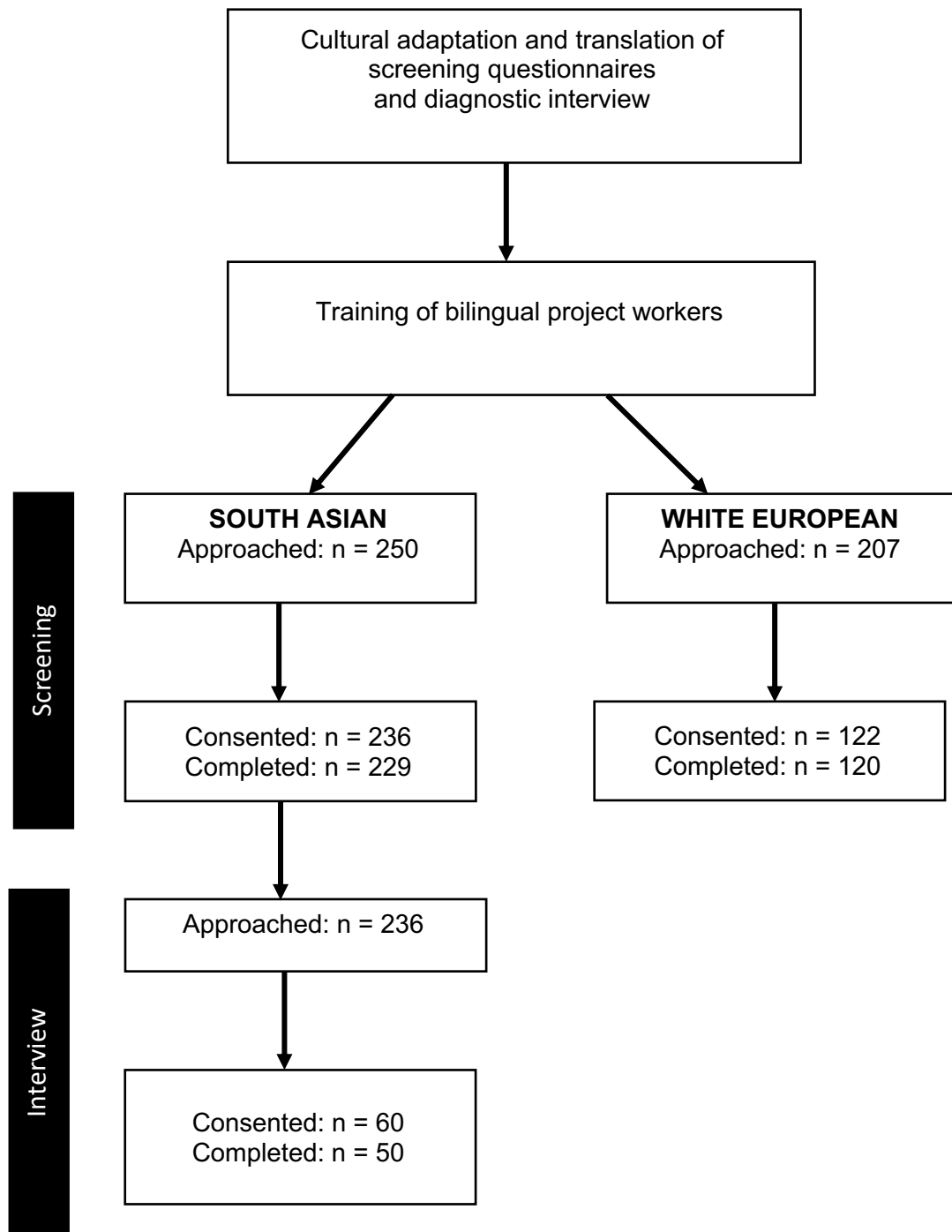

Figure S1. Study flow diagram

Supplement: S1 Fig — (PDF) [file pone.0284090.s001.pdf]

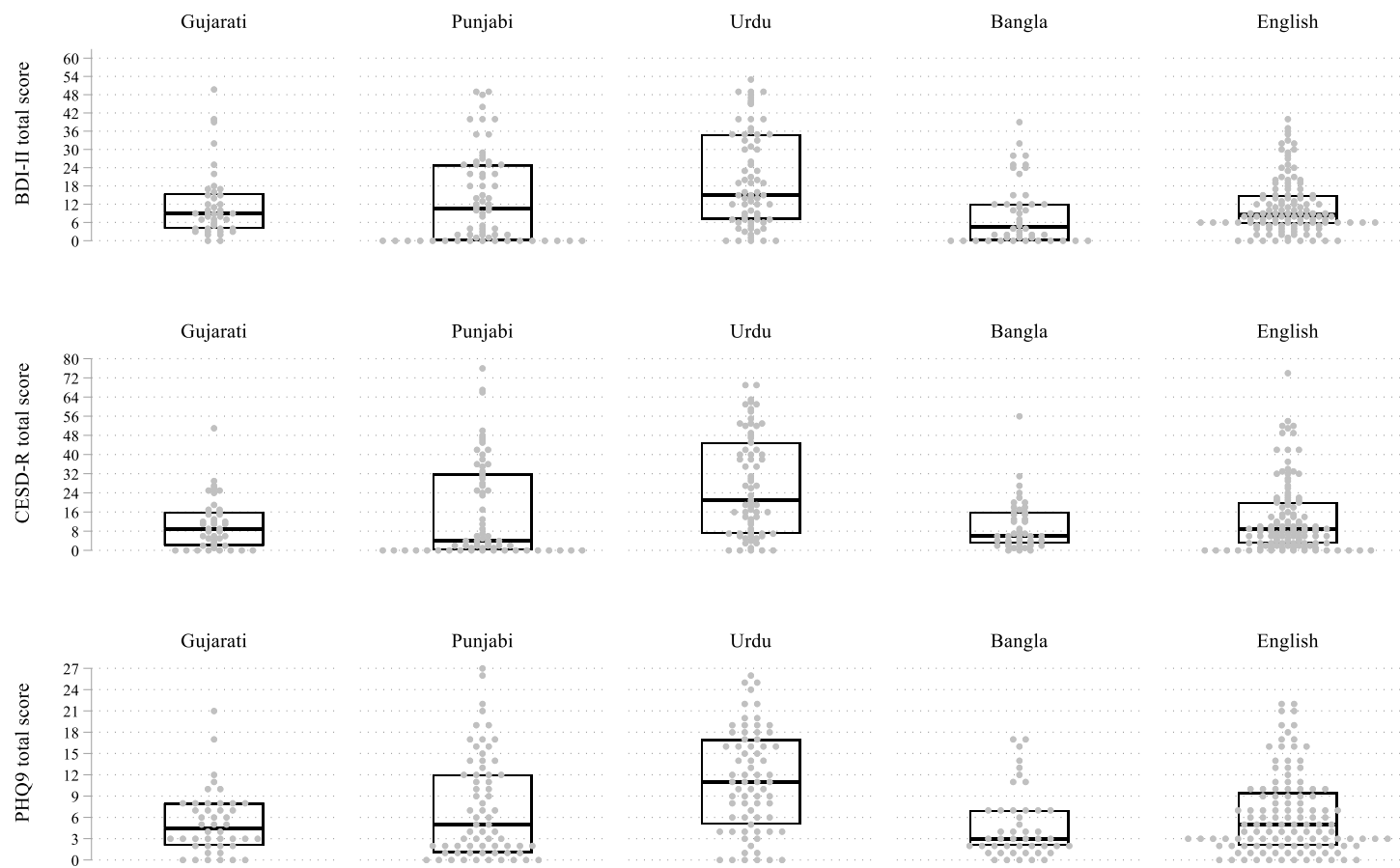

Figure S3. Total score response distributions by language group

Supplement: S3 Fig — (PDF) [file pone.0284090.s003.pdf]
