# Supplementary material for: The use of culturally adapted and translated depression screening questionnaires with South Asian haemodialysis patients in England
Source: PLoS One. 2023 Apr 7;18(4):e0284090. doi: 10.1371/journal.pone.0284090 (PMC10081747; doi:10.1371/journal.pone.0284090)
Supplement: S2 Fig — a. PHQ-9 item response patterns. b. CESD-R item response patterns. c. BDI-II item response patterns. (PDF) [file pone.0284090.s002.pdf]

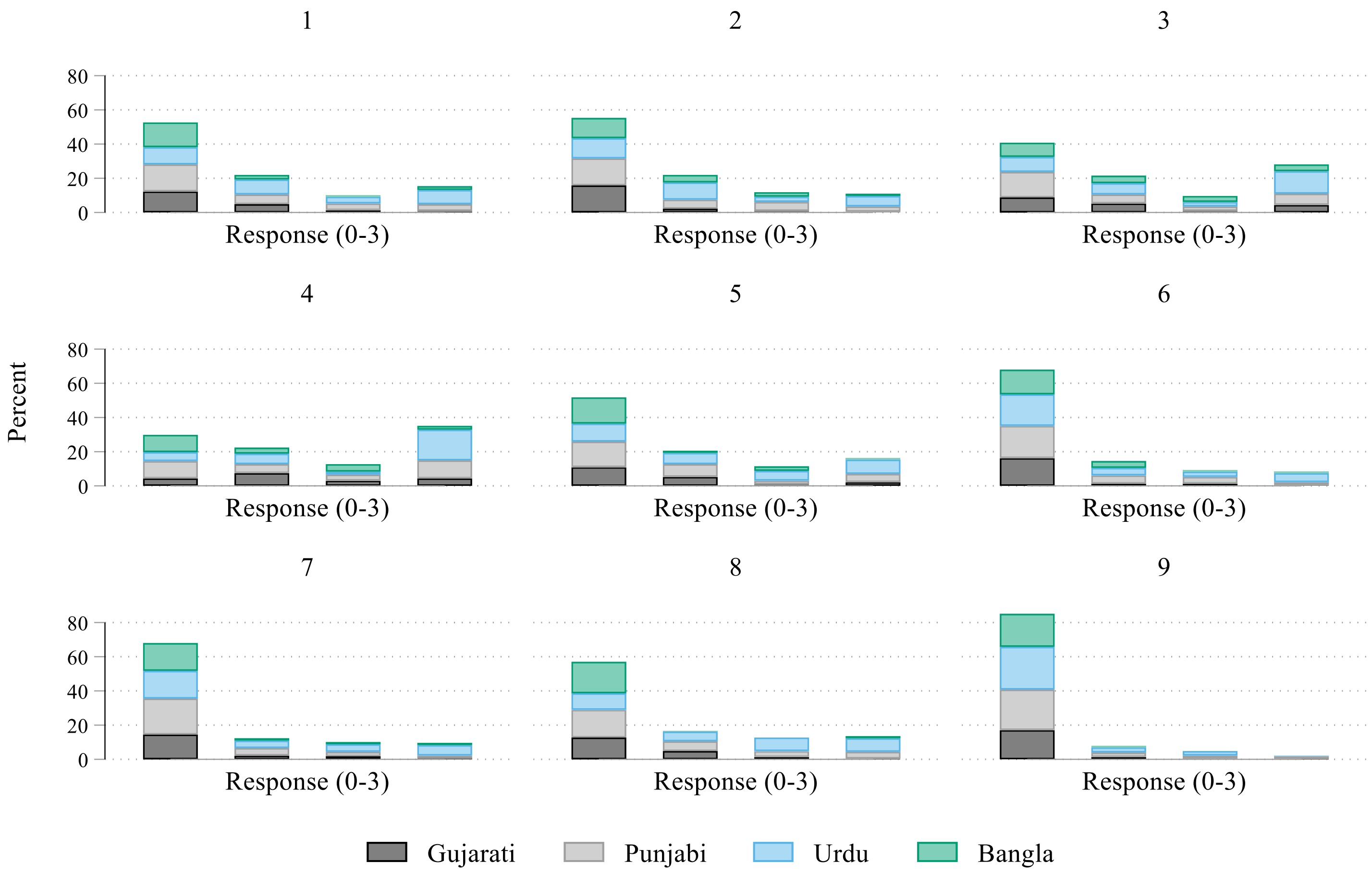

Figure S2a. PHQ-9 item response patterns

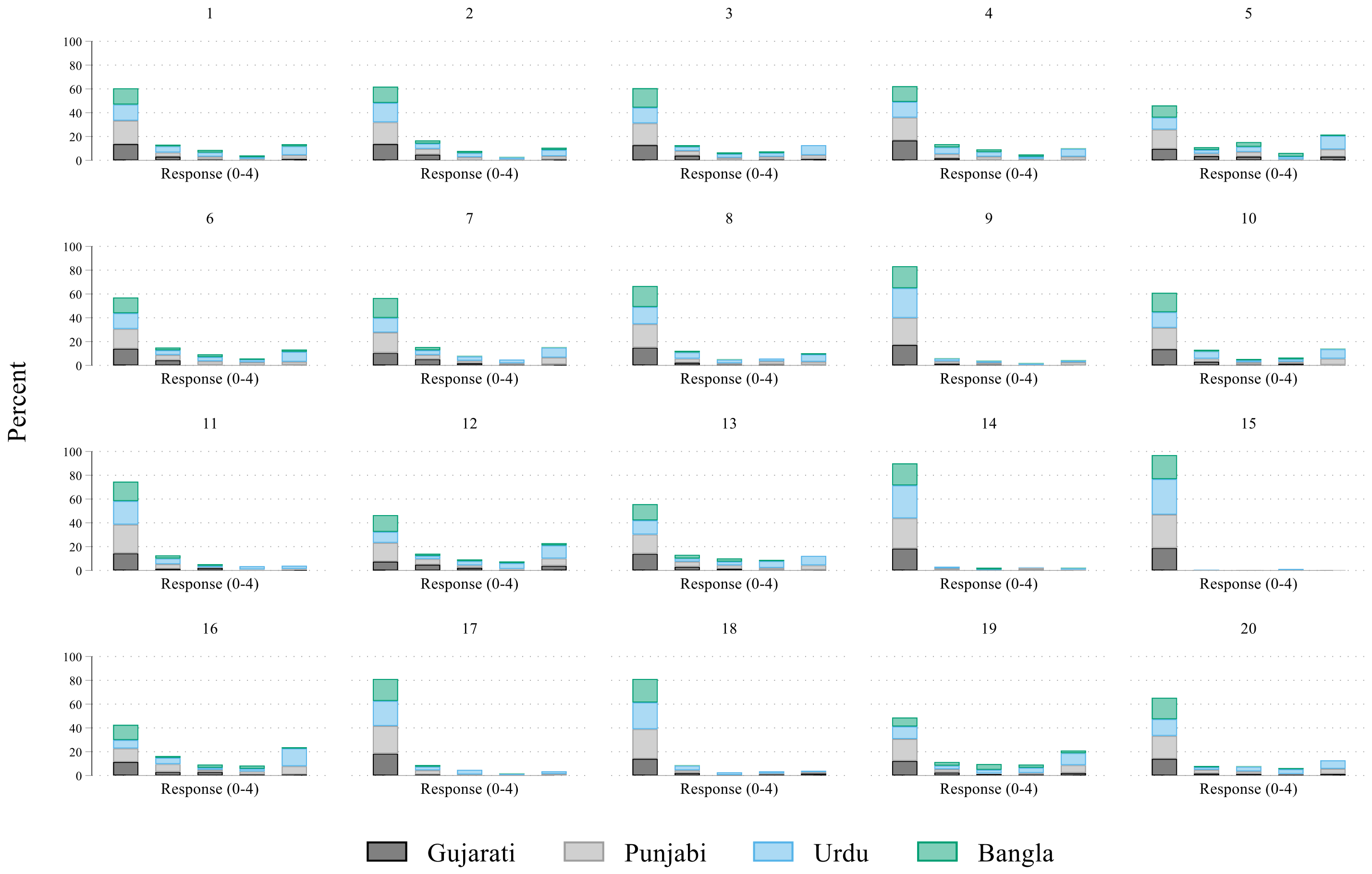

Figure S2b. CESD-R item response patterns

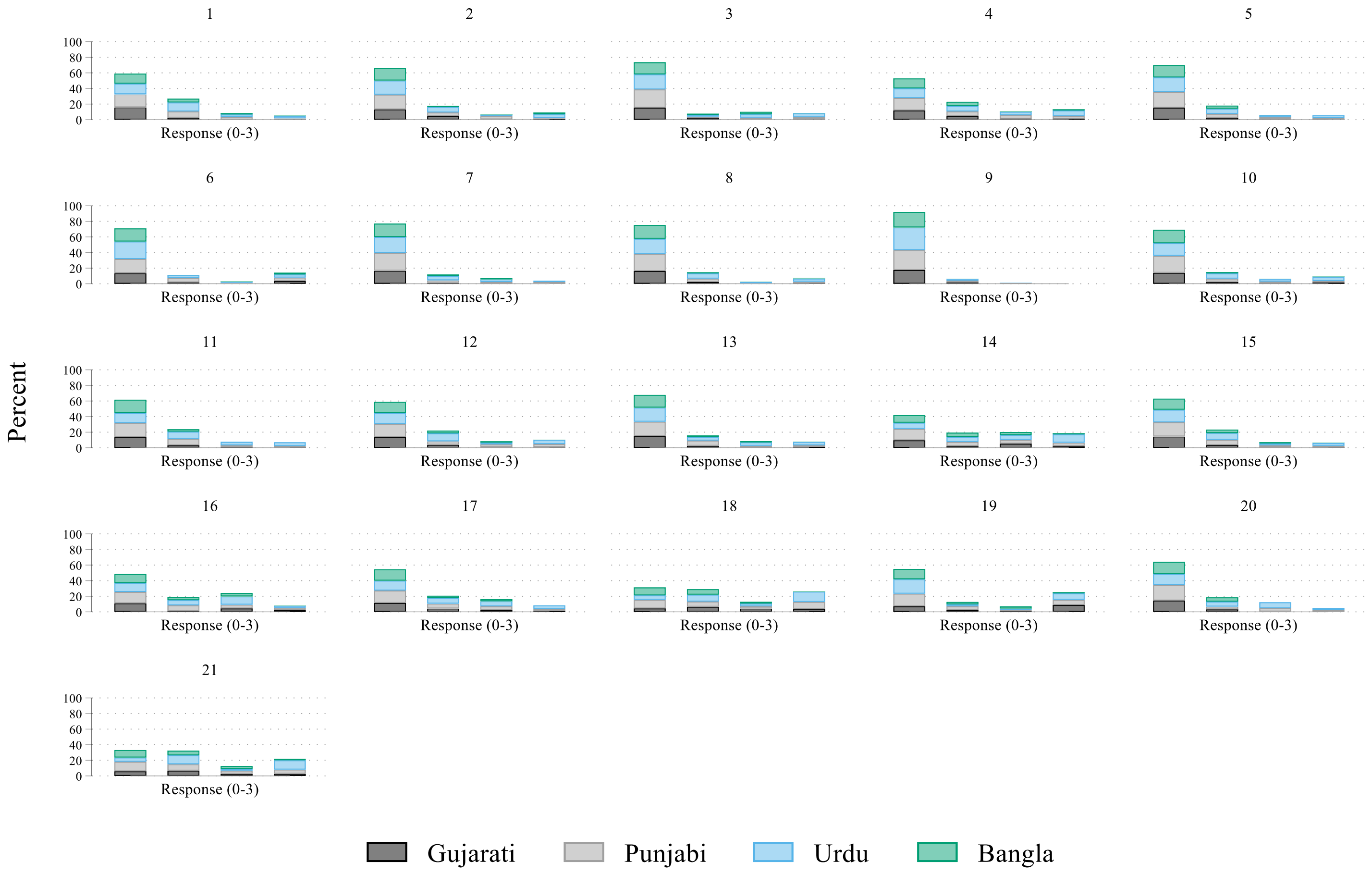

Figure S2c. BDI-II item response patterns
