# Supplementary material for: The use of culturally adapted and translated depression screening questionnaires with South Asian haemodialysis patients in England
Source: PLoS One. 2023 Apr 7;18(4):e0284090. doi: 10.1371/journal.pone.0284090 (PMC10081747; doi:10.1371/journal.pone.0284090)
Supplement: S1 Table — (PDF) [file pone.0284090.s004.pdf]

Table S1. Schedule of bilingual project worker training

All project workers had experience of working with South Asian patients in sensitive contexts. The project workers received the following training prior to working with patients.

Session	Content	Trainer
**1**	Conceptual background and aims of the study	First author (Psychologist)
**2**	Study process including working with patients and monitoring risk	First author (Psychologist); Co-author (Renal Counsellor)
**3**	Familiarisation and practice in administering questionnaires	First author (Psychologist); Co-author (Psychologist)
**4**	The CIS-R including practice interviews and coding	Co-author (Consultant Psychiatrist)
**5**	Good Clinical Practice to comply with UK National Health Service (NHS) research standards	Online course and de-briefing with co-author (Psychologist)
**6**	On-going support throughout the study including access to all training materials online, example interviews to code, regular 1-1's with study co-ordinator, quality checks and feedback on data collection	First author (Psychologist); Co-author (Psychologist); Co-author (Consultant Psychiatrist); Co-author (Consultant Nephrologist)
