# Supplementary material for: The use of culturally adapted and translated depression screening questionnaires with South Asian haemodialysis patients in England
Source: PLoS One. 2023 Apr 7;18(4):e0284090. doi: 10.1371/journal.pone.0284090 (PMC10081747; doi:10.1371/journal.pone.0284090)
Supplement: S2 Table — a. PHQ-9 factor loadings from the scalar invariant model. b. CESD-R factor loadings from the scalar invariant model. c. BDI-II factor loadings from the scalar invariant model. (PDF) [file pone.0284090.s005.pdf]

Table S2a. PHQ-9 factor loadings from the scalar invariant model

	English Loading	(SE)	Gujarati Loading	(SE)	Punjabi Loading	(SE)	Urdu Loading	(SE)	Bengali Loading	(SE)	All South Asian Loading	(SE)
PHQ_9_1	.71	(.10)	.60	(.15)	.86	(.12)	.90	(.11)	.79	(.20)	.87	(.06)
PHQ_9_2	.60	(.09)	.51	(.18)	.87	(.10)	.81	(.10)	.59	(.20)	.78	(.06)
PHQ_9_3	.75	(.09)	.43	(.18)	.77	(.14)	.72	(.13)	.67	(.16)	.71	(.07)
PHQ_9_4	.61	(.09)	.40	(.15)	.99	(.09)	.76	(.11)	.70	(.16)	.84	(.06)
PHQ_9_5	.54	(.12)	.51	(.17)	.68	(.12)	.49	(.14)	.50	(.17)	.64	(.07)
PHQ_9_6	.58	(.11)	.22	(.15)	.73	(.12)	.82	(.13)	.30	(.18)	.66	(.07)
PHQ_9_7	.64	(.11)	.62	(.14)	.63	(.12)	.78	(.13)	.36	(.18)	.68	(.07)
PHQ_9_8	.52	(.11)	.10	(.14)	.76	(.12)	.92	(.09)	.24	(.22)	.76	(.07)
PHQ_9_9	.16	(.07)	.39	(.22)	.46	(.14)	.35	(.08)	.08	(.06)	.34	(.06)
Scalar equivalence versus English		p<.001			p=.001		p=.080		p=.280		p=.070	
Scalar equivalence between South Asian											p<.001	

Table S2b. CESD-R factor loadings from the scalar invariant model

	English		Gujarati		Punjabi		Urdu		Bengali		All South Asian	
	Loading	(SE)	Loading	(SE)	Loading	(SE)	Loading	(SE)	Loading	(SE)	Loading	(SE)
CESD_R_1	.50	(.13)	.10	(.18)	.96	(.17)	.45	(.21)	.32	(.18)	.64	(.11)
CESD_R_2	.70	(.14)	.67	(.24)	1.05	(.17)	1.10	(.16)	.39	(.14)	.95	(.10)
CESD_R_3	.87	(.13)	1.01	(.16)	1.19	(.14)	1.36	(.11)	.55	(.15)	1.21	(.07)
CESD_R_4	.78	(.11)	.54	(.28)	1.10	(.16)	1.39	(.11)	.83	(.12)	1.13	(.09)
CESD_R_5	.78	(.13)	.38	(.27)	1.34	(.12)	1.21	(.14)	.47	(.12)	1.09	(.08)
CESD_R_6	.85	(.12)	.60	(.26)	1.23	(.13)	1.39	(.12)	.86	(.14)	1.21	(.08)
CESD_R_7	1.00	(.10)	.82	(.17)	1.40	(.11)	1.28	(.13)	.58	(.28)	1.22	(.08)
CESD_R_8	.76	(.12)	.80	(.22)	1.08	(.16)	1.31	(.13)	.66	(.24)	1.11	(.09)
CESD_R_9	.58	(.16)	.22	(.08)	.80	(.19)	.53	(.16)	.57	(.28)	.57	(.10)
CESD_R_10	.92	(.12)	.75	(.18)	1.18	(.14)	1.34	(.12)	.73	(.20)	1.18	(.08)
CESD_R_11	.41	(.16)	.27	(.22)	.26	(.15)	.69	(.17)	.06	(.08)	.44	(.10)
CESD_R_12	.81	(.14)	.70	(.19)	1.10	(.16)	1.23	(.12)	.62	(.18)	1.11	(.08)
CESD_R_13	.78	(.13)	.33	(.19)	1.33	(.12)	1.42	(.09)	.50	(.12)	1.22	(.07)
CESD_R_14	.26	(.13)	.24	(.13)	.58	(.20)	.38	(.14)	.59	(.27)	.42	(.10)
CESD_R_15	.02	(.02)	.02	(.03)	.21	(.15)	.15	(.10)	.	.	.13	(.07)
CESD_R_16	.89	(.11)	-.05	(.13)	1.36	(.09)	1.21	(.13)	.38	(.14)	1.17	(.08)
CESD_R_17	.55	(.14)	.01	(.03)	.63	(.20)	.73	(.15)	.40	(.22)	.60	(.10)
CESD_R_18	.50	(.17)	.35	(.30)	.28	(.13)	.43	(.15)	.00	(.01)	.32	(.09)
CESD_R_19	.94	(.15)	.27	(.31)	1.45	(.12)	1.10	(.16)	.18	(.17)	1.03	(.09)
CESD_R_20	1.08	(.12)	.93	(.19)	1.06	(.17)	1.43	(.11)	.50	(.19)	1.17	(.08)
Scalar equivalence versus English			p<.001		p=.003		p=.230		p<.001		p=.055	
Scalar equivalence between South Asian											p<.001	

Table S2c. BDI-II factor loadings from the scalar invariant model

	English		Gujarati		Punjabi		Urdu		Bengali		All South Asian	
	Loading	(SE)	Loading	(SE)	Loading	(SE)	Loading	(SE)	Loading	(SE)	Loading	(SE)
BDI_II_1	.34	(.05)	.59	(.21)	.72	(.09)	.78	(.10)	.67	(.16)	.72	(.06)
BDI_II_2	.55	(.09)	.66	(.19)	.63	(.08)	.89	(.12)	.65	(.19)	.73	(.06)
BDI_II_3	.35	(.07)	.42	(.20)	.57	(.15)	.83	(.12)	.70	(.10)	.67	(.08)
BDI_II_4	.53	(.08)	.69	(.16)	.88	(.09)	.98	(.09)	.67	(.14)	.88	(.06)
BDI_II_5	.19	(.05)	.44	(.21)	.53	(.12)	.80	(.12)	.35	(.11)	.61	(.07)
BDI_II_6	.38	(.11)	.84	(.16)	.81	(.12)	.69	(.15)	.39	(.16)	.67	(.08)
BDI_II_7	.45	(.07)	.59	(.26)	.51	(.12)	.64	(.10)	.42	(.13)	.56	(.06)
BDI_II_8	.42	(.08)	.52	(.26)	.51	(.14)	.79	(.12)	.27	(.15)	.60	(.08)
BDI_II_9	.15	(.06)	.21	(.09)	.20	(.09)	.13	(.06)	.06	(.06)	.14	(.04)
BDI_II_10	.43	(.07)	.68	(.16)	.57	(.13)	.78	(.11)	.41	(.15)	.67	(.07)
BDI_II_11	.50	(.08)	.68	(.17)	.63	(.12)	.69	(.11)	.23	(.07)	.66	(.06)
BDI_II_12	.52	(.08)	.69	(.17)	.96	(.10)	.92	(.09)	.59	(.11)	.86	(.06)
BDI_II_13	.39	(.09)	.42	(.25)	.67	(.11)	.94	(.10)	.54	(.16)	.73	(.07)
BDI_II_14	.45	(.09)	.50	(.18)	.84	(.09)	.74	(.11)	.59	(.12)	.77	(.05)
BDI_II_15	.46	(.08)	.46	(.20)	.70	(.12)	.69	(.13)	.55	(.15)	.64	(.07)
BDI_II_16	.38	(.07)	.44	(.23)	.79	(.09)	.41	(.12)	.54	(.11)	.57	(.06)
BDI_II_17	.55	(.06)	.58	(.17)	.68	(.10)	.95	(.07)	.46	(.09)	.77	(.05)
BDI_II_18	.59	(.07)	.39	(.18)	.88	(.09)	.85	(.08)	.57	(.10)	.81	(.05)
BDI_II_19	.29	(.12)	.13	(.22)	.64	(.15)	.12	(.17)	.47	(.17)	.31	(.09)
BDI_II_20	.39	(.07)	.36	(.28)	.75	(.11)	.79	(.09)	.28	(.08)	.69	(.06)
BDI_II_21	.41	(.07)	.67	(.19)	.83	(.09)	.78	(.11)	.56	(.15)	.78	(.06)
Scalar equivalence versus English			p<.001		p=.006		p<.001		p<.001		p<.001	
Scalar equivalence between South Asian											p<.001	
