# Supplementary material for: The use of culturally adapted and translated depression screening questionnaires with South Asian haemodialysis patients in England
Source: PLoS One. 2023 Apr 7;18(4):e0284090. doi: 10.1371/journal.pone.0284090 (PMC10081747; doi:10.1371/journal.pone.0284090)
Supplement: S1 File — (PDF) [file pone.0284090.s006.pdf]

Depression Screening Questionnaire Pack

Depression (Bishonnota) jachaier jonno prosnomala
ডিপ্রেশন (বিষন্নতা) যাচাইয়ের জন্য প্রশ্নমালা

Monojog deeye neecher boktobbogulo porun ebong shothik jobabtithe gool chinno din
মনোযোগ দিয়ে নীচের বক্তব্যগুলো পড়ুন এবং সঠিক জবাবটিতে গোল চিহ্ন দিন

**Whooley Questions** (হুলি কোশ্চেন)		
Goto mashey apni ki ghono ghono mon kharap, Depression (bishonnota) othoba hotashai vugeychen?<br><br>গত মাসে আপনি কি ঘন ঘন মন খারাপ, ডিপ্রেশন (বিষন্নতা) অথবা হতাশায় ভুগেছেন?	Yes<br>হ্যাঁ	No<br>না
Goto mashey apni kono kaj kortey kom agroho othoba bhalo na laga kee praiee bodh koreychen?<br><br>গত মাসে আপনি কোন কাজ করতে কম আগ্রহ অথবা ভালো না লাগা কি প্রায়ই বোধ করেছেন?	Yes<br>হ্যাঁ	No<br>না

Goto dui shoptaho dhorey apni kemon bodh korchen, neecher prosnogulo shaye somporkey. Monojog deeye ta porun ebong apnar onuvutir shathey jeti sobchey beshi milbey shaye boxtitey tik chinno din.

গত ২ সপ্তাহ ধরে আপনি কেমন বোধ করছেন, নীচের প্রশ্নগুলো সে সম্পর্কে। মনোযোগ দিয়ে তা পড়ুন এবং আপনার অনুভূতির সাথে সবচেয়ে বেশি যেটি মিলবে সেই বক্সটিতে টিক চিহ্ন দিন।

**Patient Health Questionnaire 9**<br>পেশেন্ট হেলথ কোশ্চেনিয়ার ৯	**Dui shoptaho dhore**<br>২ সপ্তাহ ধরে			
	**Motai noy**<br>মোটেই নয়	**Besh koyekdin**<br>বেশ কয়েকদিন	**Ordhekro beshi din**<br>অর্ধেকেরও বেশি দিন	**Prai protidin**<br>প্রায় প্রতিদিন
Kono kaj kortey kom agroho othoba bhalo na laga?<br>কোন কাজ করতে কম আগ্রহ অথবা ভালো না লাগা?	0<br>০	1<br>১	2<br>২	3<br>৩
Mon kharap, Depression (bishonnota) othoba hotasha bodh?<br>মন খারাপ, ডিপ্রেশন (বিষন্নতা) অথবা হতাশা বোধ?	0<br>০	1<br>১	2<br>২	3<br>৩
Ghoom ashtey othoba ghoomiey thaktey shomoshsha kingba beshi ghoom hochchey?<br>ঘুম আসতে অথবা ঘুমিয়ে থাকতে সমস্যা কিংবা বেশি ঘুম হচ্ছে?	0<br>০	1<br>১	2<br>২	3<br>৩

Klanti (hoairan) bodh koreychen othoba shokti kom pachchen?<br>ক্লান্তি (হয়রান) বোধ করেছেন অথবা শক্তি কম পাচ্ছেন?	0<br>০	1<br>১	2<br>২	3<br>৩
Khawar ruchi kom othoba beshi porimaney khachchen?<br>খাওয়ার রুচি কম অথবা বেশি পরিমাণে খাচ্ছেন?	0<br>০	1<br>১	2<br>২	3<br>৩
Nijer baparey kharap lagey- othoba nijekey bartho mone hoy kingba nijekey ba apnar poribarkey hotash (let down) korechen emon mone hoaichey?<br>নিজের ব্যাপারে খারাপ লাগে – অথবা নিজেকে ব্যর্থ মনে হয় কিংবা নিজেকে বা আপনার পরিবারকে হতাশ (লেট ডাউন) করেছেন– এমন মনে হয়েছে?	0<br>০	1<br>১	2<br>২	3<br>৩
Kono kichutey monojog detey shomossha hochchey- jemon potro-potrika portey ba television dektey?<br>কোন কিছুতে মনোযোগ দিতে সমস্যা হচ্ছে – যেমন পত্র-পত্রিকা পড়তে বা টেলিভিশন দেখতে?	0<br>০	1<br>১	2<br>২	3<br>৩
Eto aastey kotahabarta bolchen ba cholafera korchen je ta onno karo nojorey ashtey parey? Othoba er ulto- apni etotai osthir ba chonchol hoye othechen jey shavabiker thekey onek beshi norachora korchen?<br>এত আস্তে কথাবার্তা বলছেন বা চলাফেরা করছেন যে তা অন্য কারো নজরে আসতে পারে? অথবা এর উল্টো – আপনি এতোটাই অস্থির বা চঞ্চল হয়ে ওঠেছেন যে স্বাভাবিকের থেকে অনেক বেশি নড়াচড়া করছেন?	0<br>০	1<br>১	2<br>২	3<br>৩
Morey galey valo hoto emon mone hoyechey? Othoba konobhabey nijekey koshto dewar chinta mone eshechey?<br>মরে গেলে ভালো হতো এমন মনে হয়েছে? অথবা কোনভাবে নিজেকে কষ্ট দেওয়ার চিন্তা মনে এসেছে?	0<br>০	1<br>১	2<br>২	3<br>৩

**Centre for Epidemiological Studies Revised**<br>**সেন্টার ফর এপিডেমিওলজিক্যাল স্টাডিজ রিভাইজড**	**Goto shoptaho**<br>**গত সপ্তাহ**				**Go dui shoptaho dhore prai protidin**<br>গত দুই সপ্তাহ ধরে প্রায় প্রতিদিন
	**Motei na othoba ek diner kom**<br>মোটেই না অথবা একদিনের কম	**Ek-duidin**<br>১-২ দিন	**Tin-chardin**<br>৩-৪ দিন	**Panch-saatdin**<br>৫-৭ দিন	
Amar khawar ruci kom chilo<br>আমার খাওয়ার রুচি কম ছিলো	0<br>০	1<br>১	2<br>২	3<br>৩	4<br>৪
Ami amar dushchintata jherey feltey parchilamna<br>আমি আমার দুশ্চিন্তাটা ঝেড়ে ফেলতে পারছিলাম না	0<br>০	1<br>১	2<br>২	3<br>৩	4<br>৪
Kono kajey mon boshatey shomossha hochchilo<br>কোন কাজে মন বসাতে সমস্যা হচ্ছিলো	0<br>০	1<br>১	2<br>২	3<br>৩	4<br>৪
Ami depressed (bishonno) bodh koreychi<br>আমি ডিপ্রেসড (বিষন্ন) বোধ করেছি	0<br>০	1<br>১	2<br>২	3<br>৩	4<br>৪
Amar ghoomey beaghat hochchilo<br>আমার ঘুমে ব্যাঘাত হচ্ছিলো	0<br>০	1<br>১	2<br>২	3<br>৩	4<br>৪
Amar nijekey dukhi mone hoeachey<br>আমার নিজেকে দুখী মনে হয়েছে	0<br>০	1<br>১	2<br>২	3<br>৩	4<br>৪
Ami konokichu kortey parchilamna<br>আমি কোনকিছু করতে পারছিলাম না ।	0<br>০	1<br>১	2<br>২	3<br>৩	4<br>৪
Kono kichuiee amakey khushi kortey pareni<br>কোন কিছুই আমাকে খুশি করতে পারেনি	0<br>০	1<br>১	2<br>২	3<br>৩	4<br>৪
Nijekey ekta kharap manush boley mone hoyechay<br>নিজেকে একটা খারাপ মানুষ বলে মনে হয়েছে	0<br>০	1<br>১	2<br>২	3<br>৩	4<br>৪
Ami amar rojkar sokol kajkormey agroho hariey felechi<br>আমি আমার রোজকার সকল কাজকর্মে আগ্রহ হারিয়ে ফেলেছি	0<br>০	1<br>১	2<br>২	3<br>৩	4<br>৪
Ami shavabiker thekey onek beshi ghoomiyeachi<br>আমি স্বাভাবিকের থেকে অনেক বেশি ঘুমিয়েছি	0<br>০	1<br>১	2<br>২	3<br>৩	4<br>৪

Ami Khuub asthey (Dhir gotitey) cholafera korchilam boley amar mone hoieachey<br>আমি খুব আস্তে (ধীর গতিতে) চলাফেরা করছিলাম বলে আমার মনে হয়েছে	0<br>০	1<br>১	2<br>২	3<br>৩	4<br>৪
Ami osthirota/osshosthi bodh korechi<br>আমি অস্থিরতা/অস্বস্তি বোধ করেছি	0<br>০	1<br>১	2<br>২	3<br>৩	4<br>৪
Amar ichcha hoto morey jaye<br>আমার ইচ্ছে হতো মরে যাই	0<br>০	1<br>১	2<br>২	3<br>৩	4<br>৪
Ami nijekey kosto ditey cheyechi<br>আমি নিজেকে কষ্ট দিতে চেয়েছি	0<br>০	1<br>১	2<br>২	3<br>৩	4<br>৪
Ami Shob Shomoy klanto Chilam<br>আমি সব সময় ক্লান্ত ছিলাম	0<br>০	1<br>১	2<br>২	3<br>৩	4<br>৪
Ami nijeykey pochondo kortam na<br>আমি নিজেকে পছন্দ করতাম না	0<br>০	1<br>১	2<br>২	3<br>৩	4<br>৪
Kono kosto charai amar ozon onek komey gechey<br>কোন চেষ্টা ছাড়াই আমার ওজন অনেক কমে গেছে	0<br>০	1<br>১	2<br>২	3<br>৩	4<br>৪
Ghoom ashtey amar khub beshi shomossha hochchilo<br>ঘুম আসতে আমার খুব বেশি সমস্যা হচ্ছিলো	0<br>০	1<br>১	2<br>২	3<br>৩	4<br>৪
Dorkari bishoygoolotey ami mononibesh kortey parini<br>দরকারি বিষয়গুলোতে আমি মনোনিবেশ করতে পারিনি	0<br>০	1<br>১	2<br>২	3<br>৩	4<br>৪
