# Supplementary material for: The use of culturally adapted and translated depression screening questionnaires with South Asian haemodialysis patients in England
Source: PLoS One. 2023 Apr 7;18(4):e0284090. doi: 10.1371/journal.pone.0284090 (PMC10081747; doi:10.1371/journal.pone.0284090)
Supplement: S2 File — (PDF) [file pone.0284090.s007.pdf]

ડીપ્રેશનની ચકાસણી પેક **Depressionni chakaasni pek**

મહેરબાની કરીને નીચે દીધેલા બે સવાલો વાંચી અને હા અથવા ના લાગુ પડતા જવાબને સર્કલ કરો<br>Maherbani karine niche didhela be savalo vanchi ane ha athva na lagu padta jawabne circle karo		
હૂલી સવાલો Hooli savalo		
છેલ્લા એક મહિના દરમિયાન શું તમે વારંવાર ઉદાસી, ડીપ્રેશન અથવા નિરાશાના અનુભવોથી હેરાન થયા છો?<br>Chhela ek mahina darmiyaan shu tame varamvaar udaasi, depression athva niraasha na anubhavo thi heraan thaya chho?	Yes	No
છેલ્લા એક મહિનામાં શું તમને એવું લાગ્યું હતું કે કોઈ પણ કાર્ય કરવામાં ઓચ્છો રસ લાગે છે અથવા એમાં મજા નથી આવતી?<br>Chhela ek mahinama shu tamne evu lagyu hatu ke koi pan kaam karvama ochho ras lage chhe athva ema maja nathi aavti?	Yes	No

સૂચનાઓ Suchnao

છેલ્લા બે અઠવાડિયાના સમય દરમિયાન તમે શું અનુભવી રહયા છો તે વિષે તમને પૂછવા માટે નિચે સવાલો છે. મહેરબાની કરીને વિગતો બરોબર વાંચો અને તમારા અનુભવોને લાગુ પડતા જવાબો ને સર્કલ કરો.

Chhella be athvadiyana samay darmyaan tame shu anubhavi rahya chho te vishe tamne puchhva mate niche savaalo chhe. Maherbaani karine vigato barober vaancho ane tamara anubhavone lagu padta jawabo ne circle karo

પૈશંટ હેલ્થ ક્વેસ્ચેનૈર - ૯<br>**Patient Health Questionnaire-9**	છેલ્લા બે અઠવાડીયાથી Chhela be athvaadiyathi			
	જરા પણ નહી<br>Jara pan nahi	ઘણા દિવસ<br>Ghana divas	અર્ધા કરતા વધારે દિવસ<br>Ardha karta vadhare divas	લગભગદરરોજ<br>Lagbhag roj
કંઈ પણ કરવામાં ઓચ્છો રસ અથવા ઓચ્છી મજા આવવી<br>Kai pan karvama ochho ras athva ochhi maja aavavi	0	1	2	3
ગમગીન, ડીપ્રેસ્ડ અથવા નિરાશ રહેવું<br>Gamgin,depressed athva niraash rehvu	0	1	2	3
સૂવામાં તકલીફ, વચ્ચે નિંદર ઉડી જાય અથવા વધારે પડતા સૂવું<br>Suvama taklif, vache ninder udi jaai athva vadhaare padta suvu	0	1	2	3

થાક લાગતો હતો અથવા ઓછી શક્તિ હોય તેવું લાગવું<br>Thaak laagto hato athva ochhi shakti hoi tevu laagvu	0	1	2	3
ભૂખ ઓચ્છી લાગે અથવા વધારે પડતૂ ખાવુ<br>Bhookh ochhi laage athva vadhare padtu khaavu	0	1	2	3
તમારી પોતાની જાત માટે ખરાબ લાગવું અથવા એવું લાગવું કે તમે એક નિષ્ફળ વ્યક્તિ છો અને તમારી જાતને તથા તમારા કુટુંબને તમે નિરાશ કર્યા છે<br>Tamari potani jaat mate kharrab laagvu athva evu laagvu ke tame ek nishfad vyakti chho ane tamari jaatne tatha tamara kutumbne tame nirash karya chhe	0	1	2	3
ઘણા કાર્યોમાં ધ્યાન દેવામાં તકલીફ પડે જેમ કે છાપા વાચવામાં અથવા ટી.વી. જોવામાં<br>Ghana karyoma dhyaan devama taklif pade jem ke chhappa vanchvama athva t.v. jovama	0	1	2	3
એટલું ધીમું ચાલવું અથવા બોલવું કે બીજા લોકો એ નોંધ્યું હોય અથવા તેનાથી ઉલટું – એટલું બધું બેચેન કે અકળાયેલું રહેવું કે સામાન્ય કરતા વધારે પડતું હલનચલન કરવું<br>Etlu dhimu chaalvu athva bolvu ke bija loko e nondhyu hoi athva tenathi ultu - etlu badhu bechen rahevu ke saamanya karta vadhare padtu halan chalan karvu	0	1	2	3
એવા વિચારો આવે કે આના કરતા તો મરી જવું સારું અથવા તો તમને તમારી જાતને ઈજ્જા પહોંચાડવાના વિચારો આવે<br>Eva vichaaro aave ke aanaa kartaa toh mari jaavu saaru athvaa toh tamne tamari jaatne ijjaa pahonchaadvana vichaaro aave	0	1	2	3

**CESD-R**<br>છેલ્લા એક અઠવાડિયામાં તમે કેવી લાગણીઓ અનુભવી હતી અને તમારો વર્તાવ આ સમય દરમ્યાન કેવો હતો, તે તમે મહેરબાની કરીને નીચે લીસ્ટ દીધુ છે તેના થકી મને જણાવો.<br>Chhela ek athadiyama tame kevi laagnio anubhavi hati and tamaro vartaav aa samay darmyaan kevo hato te maherbaani karine tame niche list didhu chhe tena thaki mane janaavo	છેલ્લા અઠવાડીયામાં Chhela athvaadiyama				બે અઠવાડિયામાં લગભગ દરરોજ<br>Be athvadiyama lagbhag darroj
	જરા પણ નહી અથવા એક દિવસથી ઓછું<br>Jara pan nahi athva ek divas this ochhu	૧-૨ દિવસ<br>1-2 divas	૩-૪ દિવસ<br>3-4 divas	૫-૭ દિવસ<br>5-7 divas	
મારી ભૂખ ઓચ્છી થઇ ગઈ હતી<br>Maari bhookh ochhi thai gai hati	0	1	2	3	4
ઉદાસીમાંથી બહાર નીકળી શકાતું ન હતું<br>Udaasimathi bahaar nikdi shakaatu na hatu	0	1	2	3	4
મને કોઈ પણ કામમાં ધ્યાન દેવામાં મુશ્કેલી પડતી હતી<br>Mane koi pan kaam ma dhyaan devaama mushkeli padti hati	0	1	2	3	4
મને ડીપ્રેસ્ડ લાગતું હતું<br>Mane depressed lagtu hatu	0	1	2	3	4
મારી ઊંઘ બેચેન હતી<br>Maari oongh bechen hati	0	1	2	3	4
મેં ઉદાસી અનુભવી હતી<br>Mei udaasi anubhavi hati	0	1	2	3	4
મારાથી કંઈ પણ થઇ શકતું ન હતું Maara thi kai pan thai shaktu na hatu	0	1	2	3	4
મને કોઈ વસ્તુમાં ખૂશી મળતી ન હતી<br>Mane koi pan vastuma khushi madti na hati	0	1	2	3	4
મને એવું લાગતું હતું કે હું એક ખરાબ વ્યક્તિ છું<br>Mane evu laagtu hatu ke hu ek kharaab vyakti chhu	0	1	2	3	4
મેં મારી દરરોજની પ્રાવૃતિઓમાંથી રસ ગુમાવ્યો હતો	0	1	2	3	4

Mei maari darroj ni pravrutio mathi ras gumaavyo hato					
હું હંમેશ કરતા બહુ વધારે સૂતો હતો<br>Hu hamesh karta bahu vadhare suto hato	0	1	2	3	4
મને એમ લાગતું હતું કે મારું હલનચલન બહુ ધીમું હતું<br>Mane em laagtu hatu ke maaru halanchalan bahu dhimu hatu	0	1	2	3	4
મેં બેચેની અનુભવી હતી<br>Mei becheni anubhavi hati	0	1	2	3	4
મને મરી જવાની ઈચ્છા હતી<br>Mane mari javaani ichha hati	0	1	2	3	4
મને મારી જાતને ઈજા પહોંચાડવાની ઈચ્છા થતી હતી<br>Mane maari jaatne ijjaa pahonchaadvani ichha thati hati	0	1	2	3	4
હું હર વખત થાકેલો હતો<br>Hu har vakhat thakelo hato	0	1	2	3	4
હું મારી પોતાની જાતને પસંદ કરતો ન હતો<br>Hu mari potani jaatne pasand karto na hato	0	1	2	3	4
કોશિશ કર્યા વગર મારૂ વજન ઘણુ ઓચ્છું થયુ છે<br>Koshish karya vagar maaru vajan ghanu ochhu thayu chhe	0	1	2	3	4
મને સૂવામાં ઘણી તકલીફ પડી હતી Mane suvama ghani taklif padi hati	0	1	2	3	4
મારાથી મહત્વ કામો ઉપર ધ્યાન રેહતું ન હતુ<br>Marathi mahatva kaamo ooper dhyaan rahetu na hatu	0	1	2	3	4
