# Supplementary material for: The use of culturally adapted and translated depression screening questionnaires with South Asian haemodialysis patients in England
Source: PLoS One. 2023 Apr 7;18(4):e0284090. doi: 10.1371/journal.pone.0284090 (PMC10081747; doi:10.1371/journal.pone.0284090)
Supplement: S3 File — (PDF) [file pone.0284090.s008.pdf]

Depression Screening Questionnaire Pack

**Whooley Questions**		
Pichle mahine de dauraan, kee tuhaanu aksar udaas, depressed ja mayoos hon di takleef hoi hai?<br>**ਪਿਛਲੇ ਮਹੀਨੇ ਦੇ ਦੌਰਾਨ, ਕੀ ਤੁਹਾਨੂ ਅਕਸਰ ਉਦਾਸ, ਡਿਪਰੈਸਡ ਜਾਂ ਮਾਯੂਸ ਹੋਣ ਦੀ ਤਕਲੀਫ਼ ਹੋਈ ਹੈ?**	Ha<br>**ਹਾਂ**	Na<br>**ਨਾ**
Pichle mahine de dauraan, kee tuhaanu aksar kisey vi cheez ja kam karan de vich kat dilchaspi ja maza lain dee takleef hoi hai?<br>**ਪਿਛਲੇ ਮਹੀਨੇ ਦੇ ਦੌਰਾਨ, ਕੀ ਤੁਹਾਨੂੰ ਅਕਸਰ ਕਿਸੇ ਵੀ ਚੀਜ਼ ਜਾਂ ਕੰਮ ਕਰਨ ਦੇ ਵਿਚ ਘੱਟ ਦਿਲਚਸਪੀ ਜਾਂ ਮਜ਼ਾ ਲੈਣ ਦੀ ਤਕਲੀਫ਼ ਹੋਈ ਹੈ?**	Ha<br>**ਹਾਂ**	Na<br>**ਨਾ**

Hidaiyta: heta likhe hoye savaaal vich eh puchiya giya hai ki tusi apney aap nu pichle do haftiya ja pichle mahine de dauraan, kivey mahsoos karde ho. Kirpa karke het likhe savaala nu dhiaan naal padho, atey jo javaab tuhade naal mach karda ki tuhaanu kivey mahsoos hunda hai, us khaane vich tik da mark lagaou.

ਹਿਦਾਇਤਾਂ: ਹੇਠਾਂ ਲਿਖੇ ਹੋਏ ਸਵਾਲਾਂ ਵਿਚ ਇਹ ਪੁੱਛਿਆ ਗਿਆ ਹੈ ਕਿ ਤੁਸੀਂ ਆਪਣੇ ਆਪ ਨੂੰ ਪਿਛਲੇ ਦੋ ਹਫਤਿਆਂ ਜਾਂ ਪਿਛਲੇ ਨਹੀਨੇ ਦੇ ਦੌਰਾਨ ਕਿਵੇਂ ਮਹਿਸੂਸ ਕਰਦੇ ਹੋ। ਕਿਰਪਾ ਕਰਕੇ ਹੇਠ ਲਿਖੇ ਸਵਾਲਾਂ ਨੂੰ ਧਿਆਨ ਨਾਲ ਪੜੋ, ਅਤੇ ਜੋ ਜਵਾਬ ਤੁਹਾਡੇ ਨਾਲ ਮੈਚ ਕਰਦਾ ਕਿ ਤੁਹਾਨੂੰ ਕਿਵੈਂ ਮਹਿਸੂਸ ਹੁੰਦਾ ਹੈ, ਉਸ ਖਾਨੇ ਵਿਚ ਟਿੱਕ ਦਾ ਮਾਰਕ ਲਗਾਉ।

**PHQ-9**	**Pichle do haftiya vich**<br>**ਪਿਛਲੇ ਦੋ ਹਫਤਿਆਂ ਵਿਚ**			
	**Bilkul nahi**<br>**ਬਿਲਕੁਲ ਨਹੀਂ**	**Kayi din**<br>**ਕਈ ਦਿਨ**	**Adhe din to ziada**<br>**ਅੱਧੇ ਦਿਨ ਤੋਂ ਜ਼ਿਆਦਾ**	**Takri-ban har roz**<br>**ਤਕਰੀਬਨ ਹਰ ਰੋਜ਼**
Kise kam vich kat dilchaspi ja maza?<br>**ਕਿਸੇ ਕੰਮ ਕਰਨ ਵਿਚ ਘੱਟ ਦਿਲਚਸਪੀ ਜਾਂ ਮਜ਼ਾ?**	0	1	2	3
Udaas, depressed ja mayoos mahsoos hona?<br>**ਉਦਾਸ, ਡਿਪਰੈਸਡ ਜਾਂ ਮਾਯੂਸ ਮਹਿਸੂਸ ਹੋਣਾ?**	0	1	2	3
Neend aun vich ja sutte rahain vich mushkil, ja bohut ziaada sutte rahaina?<br>**ਨੀਂਦ ਆਉਣ ਵਿਚ ਜਾਂ ਸੁੱਤੇ ਰਹਿਣ ਵਿਚ ਮੁਸ਼ਕਲ, ਜਾਂ ਬਹੁਤ ਜ਼ਿਆਦਾ ਸੁੱਤੇ ਰਹਿਣਾ?**	0	1	2	3

Thakavat mahsoos karni ja kise vi kam karan vich koyi taakat di kami?<br>**ਥੱਕਾਵਟ ਮਹਿਸੂਸ ਕਰਨੀ ਜਾਂ ਕਿਸੇ ਵੀ ਕੰਮ ਕਰਨ ਵਿਚ ਕੋਈ ਤਾਕਤ ਦੀ ਕਮੀ?**	0	1	2	3
Bhuk kat lagni ja bohuta khaana?<br>**ਭੁੱਖ ਘੱਟ ਲਗਣੀ ਜਾਂ ਬਹੁਤਾ ਖਾਣਾ?**	0	1	2	3
Apne bare bura mahsoos karna - ki tusee asafal viakti ho atey apne aap nu ja apne parivar nu nirash kita hai?<br>**ਆਪਣੇ ਬਾਰੇ ਬੁਰਾ ਮਹਿਸੂਸ ਕਰਨਾ – ਕਿ ਤੁਸੀਂ ਅਸਫ਼ਲ ਵਿਆਕਤੀ ਹੋ ਅਤੇ ਆਪਣੇ ਆਪ ਨੂੰ ਜਾਂ ਆਪਣੇ ਪਰਿਵਾਰ ਨੂੰ ਨਿਰਾਸ਼ ਕੀਤਾ ਹੈ?**	0	1	2	3
Akhbaar padhan vich ja television dekhan vich atey is tarha diya cheesa vich dhiaan takaun vich mushkil aundi hai?<br>**ਅਖਬਾਰ ਪੜ੍ਹਣ ਜਾਂ ਟੈਲੀਵਿਜਨ ਦੇਖਣ ਵਿਚ ਅਤੇ ਇਸ ਤਰ੍ਹਾਂ ਦੀਆਂ ਚੀਜ਼ਾਂ ਵਿਚ ਧਿਆਨ ਟਕਾਉਣ ਵਿਚ ਮੁਸ਼ਕਲ ਆਉਂਦੀ ਹੈ?**	0	1	2	3
Ehna hauli chalna ja bolna ki doosriya de nazar vich aa sakde si? Ja is de ult - bechaini ja beyaraami honi jis karke aam taur to bohut ziaada hilna julna?<br>**ਇੰਨਾ ਹੌਲੀ ਚਲਣਾਂ ਜਾਂ ਬੋਲਣਾ ਕਿ ਦੂਸਰਿਆਂ ਦੇ ਨਜ਼ਰ ਵਿਚ ਆ ਸਕਦੇ ਸੀ? ਜਾਂ ਇਸ ਦੇ ਉਲਟ – ਬੇਚੈਨੀ ਜਾਂ ਬੇਅਰਾਮੀ ਹੋਣੀ ਜਿਸ ਕਰਕੇ ਆਮ ਤੌਰ ਤੋਂ ਬਹੁਤ ਜ਼ਿਆਦਾ ਹਿਲਣਾ ਜੁਲਣਾ?**	0	1	2	3
Eho jihe khaiyal aune ki mar jana changa hai ja apne aap nu kise tarha di chot pachauni?<br>**ਇਹੋ ਜਿਹੇ ਖਿਆਲ ਆਉਣੇ ਕਿ ਮਰ ਜਾਣਾ ਚੰਗਾ ਹੈ ਜਾਂ ਆਪਣੇ ਆਪ ਨੂੰ ਕਿਸੇ ਤਰ੍ਹਾਂ ਦੀ ਚੋਟ ਪਹੁੰਚਾਉਣੀ?**	0	1	2	3

**CESD-R**	**Pichle Hafte**<br>**ਪਿਛਲੇ ਹਫਤੇ**				**Takriban har roz do haftiya layi**<br>**ਤਕਰੀਬਨ ਹਰ ਰੋਜ਼ ਦੋ ਹਫਤਿਆਂ ਲਈ**
	**Bilkul nahi ja ek din nalo kat**<br>**ਬਿਲਕੁਲ ਨਹੀਂ ਜਾਂ ਇਕ ਦਿਨ ਨਾਲੋਂ ਘੱਟ**	**1-2 din**<br>**1-2 ਦਿਨ**	**3-4 din**<br>**3-4 ਦਿਨ**	**5-7 din**<br>**5-7 ਦਿਨ**	
Menu bhuk kat lagdi si<br>**ਮੈਨੂੰ ਭੁੱਖ ਘੱਟ ਲਗਦੀ ਸੀ**	0	1	2	3	4
Mai udaasi to chhutkara nahi kar sakda/sakdi si	0	1	2	3	4

ਮੈਂ ਉਦਾਸੀ ਤੋਂ ਛੁਟਕਾਰਾ ਨਹੀਂ ਪਾ ਸਕਦਾ/ਸਕਦੀ ਸੀ					
Menu apne kam te dhiaan rakhan vich mushkil aundi si<br>**ਮੈਨੂੰ ਆਪਣੇ ਕੰਮ ਤੇ ਧਿਆਨ ਰੱਖਣ ਵਿਚ ਮੁਸ਼ਕਲ ਆਉਂਦੀ ਸੀ**	0	1	2	3	4
Mai depression mahsoos karda/kardi si<br>**ਮੈਂ ਡਿਪਰੈਸ਼ਨ ਮਹਿਸੂਸ ਕਰਦਾ/ਕਰਦੀ ਸੀ ਸੀ**	0	1	2	3	4
Meri neend beychain si<br>**ਮੇਰੀ ਨੀਂਦ ਬੇਚੈਨ ਸੀ**	0	1	2	3	4
Menu udaasi mahsoos hoyi<br>**ਮੈਨੂੰ ਉਦਾਸੀ ਮਹਿਸੂਸ ਹੋਈ**	0	1	2	3	4
Mere kolo kujh nahi hunda si<br>**ਮੇਰੇ ਕੋਲੋਂ ਕੁਝ ਨਹੀਂ ਹੁੰਦਾ ਸੀ**	0	1	2	3	4
Koyi chees to menu khushi nahi mildi si<br>**ਕੋਈ ਚੀਜ ਤੋਂ ਮੈਨੂੰ ਖੁਸ਼ੀ ਨਹੀਂ ਮਿਲਦੀ ਸੀ**	0	1	2	3	4
Menu lagda si ki mai bura insaan ha<br>**ਮੈਨੂੰ ਲਗਦਾ ਸੀ ਕਿ ਮੈਂ ਬੁਰਾ ਇਨਸਾਨ ਹਾਂ**	0	1	2	3	4
Mere aam kama vich meri dilchaspi nahi rahi<br>**ਮੇਰੇ ਆਮ ਕੰਮਾਂ ਵਿਚ ਮੇਰੀ ਦਿਲਚਸਪੀ ਨਹੀਂ ਰਹੀ**	0	1	2	3	4
Mai aam taur naalo bohut ziaada saunda/saundi si<br>**ਮੈਂ ਆਮ ਤੌਰ ਨਾਲੋਂ ਬਹੁਤ ਜ਼ਿਆਦਾ ਸੌਂਦਾ/ਸੌਂਦੀ ਸੀ**	0	1	2	3	4
Menu lagda si ki mai bohut hauli hilda/hildi si<br>**ਮੈਨੂੰ ਲਗਦਾ ਸੀ ਕਿ ਮੈਂ ਬਹੁਤ ਹੌਲੀ ਹਿਲਦਾ/ਹਿਲਦੀ ਸੀ**	0	1	2	3	4
Menu bechaini mahsoos hundi si<br>**ਮੈਨੂੰ ਬੇਚੈਨੀ ਮਹਿਸੂਸ ਹੁੰਦੀ ਸੀ**	0	1	2	3	4
Menu maran di ichha hundi si<br>**ਮੈਨੂੰ ਮਰਨ ਦੀ ਇੱਛਾ ਹੁੰਦੀ ਸੀ**	0	1	2	3	4
Mai aapne aap nu chot pachauna chaunda/chaundi si<br>**ਮੈਂ ਆਪਣੇ ਆਪ ਨੂੰ ਚੋਟ ਪਹੁੰਚਾਉਣਾ ਚਾਹੁੰਦਾ/ਚਾਹੁੰਦੀ ਸੀ**	0	1	2	3	4
Mai har vele thakka rahinda si<br>**ਮੈਂ ਹਰ ਵੇਲੇ ਥੱਕਾ ਰਹਿੰਦਾ/ ਰਹਿੰਦੀ ਸੀ**	0	1	2	3	4
Mai apne aap nu pasand nahi karda si<br>**ਮੈਂ ਆਪਣੇ ਆਪ ਨੂੰ ਪਸੰਦ ਨਹੀਂ ਕਰਦਾ/ਕਰਦੀ ਸੀ**	0	1	2	3	4

Koshish karan to begair mera bohut bhaar kat giya si<br>**ਕੋਸ਼ਿਸ਼ ਕਰਨ ਤੋਂ ਬਗੈਰ ਮੇਰਾ ਬਹੁਤ ਭਾਰ ਘੱਟ ਗਿਆ ਸੀ**	0	1	2	3	4
Menu saun vich bohut ziaada mushkil hundi si<br>**ਮੈਨੂੰ ਸੌਣ ਵਿਚ ਬਹੁਤ ਜ਼ਿਆਦਾ ਮੁਸ਼ਕਲ ਹੁੰਦੀ ਸੀ**	0	1	2	3	4
Mai zaroori cheeza te dhiaan nahi de sakda/sakdi si<br>**ਮੈਂ ਜ਼ਰੂਰੀ ਚੀਜਾਂ ਤੇ ਧਿਆਨ ਨਹੀਂ ਦੇ ਸਕਦਾ/ਸਕਦੀ ਸੀ**	0	1	2	3	4
