# Supplementary material for: The use of culturally adapted and translated depression screening questionnaires with South Asian haemodialysis patients in England
Source: PLoS One. 2023 Apr 7;18(4):e0284090. doi: 10.1371/journal.pone.0284090 (PMC10081747; doi:10.1371/journal.pone.0284090)
Supplement: S4 File — (PDF) [file pone.0284090.s009.pdf]

Depression Screening Questionnaire Pack

Whooley Questions		
**Guzishta mahiney ke doraan, aap ko gahri udaasi, depressed honay, ya naumeedi ki wajah se kia aap ko aksar mushkil paish aai hai?**<br>گزشتہ مہینے کے دوران، آپ کو اداسی، ڈیپریسڈ ہونے، یا ناامیدی کی وجہ سے کیا آپ کو اکثر مسکل پیش آئی ہے؟	Yes	No
**Guzishta mahiney ke doraan, kia aapko kisi cheez ya kaam mein dilchaspi ya mazza kam honay ki wajah se takleef hui hai?**<br>گزشتہ مہینے کے دوران کیا آپ کو کسی چیز یا کام میں دلچپی یا مزہ نہ ہونے کی وجہ سے تکلیف ہوئی ہے	Yes	No

Hidayat: madarja zail sawalaat pichley do haftey ke doraan aapke ahsasaat ke barey mein hain. Barai meherbani is bayaan ko dehaan se parhein, aur apney jawabaat mutalqa bayaan, Jo ke aap ke haal ko behtar tareeqey se bayaan karta hai, is ke saath likhey huey hindsay par daira laga ke batain.

ہدایات: مدرجہ ذیل سوالات پچھلے دو ہفتے کے دوران آپ کے احساسات کے بارے میں ہیں۔ برائے مہربانی اس بیان کو دیھان سے پڑھیں، اور اپنے جوابات مطلقہ بیان، جو کے آپ کے حال کو بہتر طریقے سے بیان کرتا ہے، اس کے ساتھ لکھے ہوئے ہندسے پر دائرہ لگا کے بتائیں۔

PHQ-9	Over the last 2 weeks<br>پچھلے دو ہفتے کے دوران			
	Not at all<br>بالکل نہیں	Several days<br>کئی دن	More than half the days<br>آدھے سے زیادہ	Nearly every day<br>تقریباً روزانہ
Kuch Karney mein kam dilchaspi hoti hai ya kam mazza aata hai<br>کچھ کرنے میں کم دلچپی ہوتی ہے یا کم مزا آتا ہے	0	1	2	3
Afsurda, depressed ya naumeed mehsoos karna<br>افسردہ، ڈیپریسڈ یا ناامیدی محسوس کرنا	0	1	2	3
Soney ya soye rahney mein mushkil hona, ya bohat zayada sona.<br>سونے یا سوئے رہنے میں مسکل ہونا، یا بہت زیادہ سونا۔	0	1	2	3

Thakaan mahsoos karna ya kam taaqat hona<br><br>ھکان محسوس کرما یا کم طاقت ہوما۔	0	1	2	3
Bhook mein kami ya bohat zayada khaana<br><br>بھوک میں کمی یا بہت زیادہ کھاما۔	0	1	2	3
Apney mutalaq bura mahsoos karna - ya yeh ke aap nakaam hain ya aapne Khud ko ya apney khandaan ko mayoos Kia hai?<br><br>اپنے معلق برا محسوس کرنا- یا یہ کہ آپ ماکام ہیں یا آپ<br>ے حود کو یا اپے حامدان کو مایوس کیا ہے-	0	1	2	3
Kisi kaam mein tawajjo markooz karney mein mushkil hona, jaise ke akhbaar parhna ya tv dekhna?<br>کسی کام میں وجہ مرکوز کرے میں مسکل ہونا، جیسے کہ<br>احبار پڑھا یا ی وی دیکھنا۔	0	1	2	3
Itna aahista harkat ya baat karna jisey doosrey log mahsoos kar saktey hoon? Ya is ka ulat.<br>Itna zayada hillna julna ya bearaami mahsoos karna ke aap ma'amool se zayada harkat karey.<br><br>انا آہستہ حرکت یا بات کرما جسے دوسرے لوگ محسوس کر<br>سکے ہوں؟ یا اس کا الٹ۔<br>اتنا زیاہ ہلنا جلنا یا بے آرامی محسوس کرما کہ آپ معمول<br>سے زیادہ حرکت کریں۔	0	1	2	3
Is kisam ke khayalaat aana ke aap ka mar jaana hi behtar hai ya khood ko kissi tareeqey se takleef pohchaney ke barey mein sochna.<br>اس قسم کے حیلات آما کہ آپ کا مرجاما ہی بہتر ہے یا حود<br>کو کسی طریقے سے لکلیف پہچاے کے بارے میں<br>سوچا۔	0	1	2	3

CESD-R	Last Week				Pichley do haftey mein taqreeban rozana
	**Bilkul bhi nahi ya aik din se kam**<br>بالکل بھی نہیں یا ایک دن سے کم	**Aik se do din**<br>1-2<br>دن	**Teen se chaar din**<br>**3-4**<br>دن	**Paanch se saat din**<br>5-7<br>دن	پچھلے دو ہفتے میں تقریباً روزانہ
Mujhey bhook kam lagti thi<br>مجھے بھوک کم لگتی تھی	0	1	2	3	4
Mujhey udaasi se chutkaara nahi mil raha tha<br>مجھے اداسی سے چھٹکارا نہیں مل رہا تھا	0	1	2	3	4
Mujhey apney kaam par dehaan deney mein mushkil ho rahi thi<br>مجھے اپنے کام پر دھیان دینے میں مشکل ہو رہی تھی۔	0	1	2	3	4
Mein depressed mehsoon kar raha tha / thi<br>میں ڈیپریسڈ محسوس کر رہا تھا / رہی۔	0	1	2	3	4
Meir neend mein bechaini thi<br>میری نیند میں بےچینی تھی۔	0	1	2	3	4
Mujhey udaasi mehsoos ho rahi thi<br>مجھے اداسی محسوس ہو رہی تھی	0	1	2	3	4
Mujh se kuch bhi nahi ho pa raha tha<br>مجھ سے کچھ بھی نہیں ہو پا رہا تھا۔	0	1	2	3	4
Mujhey kissi cheez mein khushi nahi mil rahi thi<br>مجھے کسی چیز میں خوشی نہیں مل رہی تھی	0	1	2	3	4
Mujhey mehsoos hota tha ke mein aik bura insaan hoon<br>مجھے محسوس ہوتا تھا کہ میں برا انسان ہوں۔	0	1	2	3	4
Meri roz marra ke mashaghil mein dilchaspi nahi rahi<br>میری روزمرہ کے مشاغل میں دلچسپی نہیں رہی۔	0	1	2	3	4

Mera sona ma'amool se kaafi ziada tha<br>میرا سوا معمول سے کافی زیادہ تھا۔	0	1	2	3	4
Mujhey mehsoos hua ke mera hillna julna bohat aahista tha<br>مجھے محسوس ہوا کہ میرا ہلنا جلنا بہت آہستہ تھا۔	0	1	2	3	4
Mujhey bechaini mehsoos ho rahi thi<br>مجھے بے چینی محسوس ہو رہی تھی۔	0	1	2	3	4
Mujhey marney ki khawahish thi<br>مجھے مرنے کی خواہش تھی۔	0	1	2	3	4
Mein apney aap ko chot pohchana chahta tha / chahti thi<br>میں اپنے آپ کو چوٹ پہچانا چاہی تھی / چاہتا تھا۔	0	1	2	3	4
Mein har waqt thakka hua rahta tha / thi<br>میں ہر وقت تھکا ہوا رہتا تھا / تھی	0	1	2	3	4
Mujhey apna aap acha nahi lag raha tha<br>مجھے اپنا آپ اچھا نہیں لگ رہا تھا	0	1	2	3	4
Baghair koshish kiye mera kaafi wazan kam hua hai<br>بغیر کوشش کیے میرا کافی وزن کم ہوا ہے۔	0	1	2	3	4
Mujhey soney mein bohat mushkil pesh aarahi thi<br>مجھے سوے میں بہت مسکل پیش آرہی تھی۔	0	1	2	3	4
Mein zaroori cheezon par dehaan nahi de sakta tha / sakti thi<br>میں صروری چیزوں پر دیہان نہیں دے سکتا تھا / سکتی تھی۔	0	1	2	3	4
